# Postoperative pain after colorectal surgery

**DOI:** 10.1007/s00384-020-03580-4

**Published:** 2020-04-21

**Authors:** Margaretha Lindberg, Oskar Franklin, Johan Svensson, Karl A. Franklin

**Affiliations:** 1 Department of Surgical and Perioperative Sciences, Surgery, Umeå University, SE-901 85 Umeå, Sweden; 2 Department of Statistics, Umeå School of Business, Economics and Statistics, Umeå University, Umeå, Sweden

**Keywords:** Postoperative pain, Colorectal surgery, Numeric rating scale, Minimally invasive surgery, Risk factors

## Abstract

**Purpose:**

Postoperative pain is a keystone in perioperative programs, as pain negatively impacts recovery. This study aimed to evaluate pain after elective colorectal surgery and to identify risk factors for postoperative pain.

**Methods:**

This prospective cohort study comprised consecutive patients undergoing elective colorectal surgery within the Enhanced Recovery after Surgery (ERAS) perioperative program between March 2013 and April 2017. The numeric rating scale (NRS) was used to estimate maximum pain. Logistic regression was used to model associations with the type of surgery, age, gender, and comorbidities.

**Results:**

The cohort comprised 434 of 459 eligible patients. On the day of surgery to postoperative day 3, 50–64% of patients reported moderate to severe pain (NRS 4–10). Postoperative pain was similar for open and minimally invasive rectal surgery, while patients undergoing minimally invasive colonic surgery experienced more pain on the day of surgery and less pain on postoperative days 2 and 3 vs. open colonic surgery. Younger age was associated with more pain every postoperative day and by 0.7 NRS/10 years (95% CI 0.5–0.9, *P* < 0.001) on the day of surgery, while having diabetes type 2 was associated with less postoperative pain by − 1.3 NRS (95% CI − 2.4 to − 0.2) on the day of surgery.

**Conclusions:**

The majority, and young patients in particular, experience moderate to severe pain after open and minimally invasive colorectal surgery, despite following ERAS perioperative program. There is a need for effective and individualized analgesia after colorectal surgery, since the individual pain response to surgery is difficult to predict.

## Introduction

Pain after surgery is a major concern for patients, especially when it is undermanaged [1]. Postoperative pain delays mobilization and oral intake after surgery, as well as increasing the risk of chronic pain after surgery [2, 3]. Colorectal cancer is the third most common cancer, with 1.2 million new cases a year, the majority of which undergo surgery [4].

A numeric rating scale (NRS) scores pain from 0 to 10. A tolerable threshold for pain is estimated at NRS = 3, and patients scoring NRS > 4, i.e., moderate and severe pain, are therefore in need of extra analgesia [5, 6]. Reported risk factors for increased postoperative pain in general include a high American Society of Anesthesiologist classification (ASA class), young age, preoperative pain, female gender, and the anatomic location of surgery [7–12]. There is, however, a lack of prospective cohort studies of pain after colorectal surgery and the comparison of pain after open and minimally invasive surgery. Enhanced Recovery after Surgery (ERAS) guidelines on colorectal surgery recommend opioid-sparing multimodal analgesia, with paracetamol as a basic part, in combination with epidural analgesia after open surgery [13–17].

This study aimed to quantify pain after elective colorectal surgery and to identify risk factors for postoperative pain.

## Methods

### Ethical approval

The study protocols were approved by the Umeå University ethical board and all the patients gave their informed written consent.

### Study design

This prospective cohort study comprised all consecutive patients undergoing elective colorectal surgery at Umeå University Hospital in Sweden between March 2013 and April 2017. Patient data were prospectively registered in the ERAS interactive audit system database, except for high-sensitive C-reactive protein (CRP) which was retrieved from patient records. Patients were excluded if the numeric rating scale (NRS) was not scored. The included procedures were open or minimally invasive colonic surgery, anterior rectum resection, and abdominal perineal excision including the whole rectum and anus. All the patients were treated according to ERAS guidelines [14–16].

### Primary outcome measurement

The primary outcome measurement was the NRS, graded from 0 to 10, where 0 = no pain and 10 = the worst imaginable pain [18]. Moderate pain was defined as NRS 4–6 and severe pain as NRS 7–10. The patients were questioned by nurses each morning on four postoperative days to score the maximum pain during the previous 24 h using the NRS.

### Postoperative analgesia

The target for postoperative analgesia was an NRS score of 3 or less. The protocol included acetaminophen to all patients during the whole postoperative period in a dose of 4 g daily, while nonsteroidal anti-inflammatory drugs were not given due to the risk of anastomotic leakage [19]. Thoracic epidural analgesia was given to patients undergoing open colonic surgery until day 2, while those undergoing open rectal resection received it until postoperative day 4, followed by long-acting opioids orally, twice a day, and short-acting opioids orally on demand. The epidural analgesia was inserted before the induction of general anesthesia and Breivik’s mixture of analgesia was given. It comprised 1 mg/ml of bupivacaine, 2 μg/ml of fentanyl, and 2 μg/ml of epinephrine at a rate of 3–12 ml an hour [20].

The protocol for minimally invasive surgery, i.e., laparoscopic and robot-assisted surgery, included local anesthesia in the incisions and spinal anesthesia during rectal surgery. It also included short-acting opioids intravenously on demand on the day of surgery. Long-acting opioids were given orally twice daily after minimally invasive surgery on postoperative day 1, while short-acting opioids were given orally on demand.

### Clinical variables

Baseline clinical variables were assessed preoperatively. They included age, gender, body mass index (BMI), smoking, a diabetes mellitus diagnosis, and ASA class. High-sensitivity C-reactive protein (CRP) was measured every postoperative morning. The postoperative course was registered prospectively and included preoperative oncological treatment, a histopathologically verified cancer diagnosis, the length of hospital stay and complications, including any complication, Clavien-Dindo 3b or more and anastomotic leakage within 30 days after surgery [21].

### Power calculation

A power of 80% and a significance level of 5% were used and NRS scores were assumed to vary with a standard deviation of 3. A sample size of 80 to 160 patients was estimated if a continuous predictor (e.g., age, BMI) was able to explain about 5 to 10% of the variation in NRS. A sample size of about 100 patients was estimated to detect a mean change in NRS score of one unit for nominal variables that split the set into equally sized parts (e.g., gender) and about 250 patients for unequal splits (e.g., smokers, complications, and different operations). Assuming 30% missing data, we estimated a need for 120 to 350 observations.

### Statistical analysis

Stata (version 14) and SPSS (version 24) were used for the statistical analyses. A two-sided *t* test was used to compare differences in NRS between groups. Univariable (unadjusted) and multivariable (adjusted) linear regression analyses were used to analyze how the NRS for maximum pain depended on a number of predictor variables. The chi-squared test was used to analyze categorical differences in pain. Throughout the report, we used a significance level of 5% and a two-sided hypothesis test. Pearson’s correlation was used to analyze correlations between the NRS scores for different days. Missing data for the NRS pain score were assumed to be missing completely at random, since the occurrence of missing data depended on single nurses and was unrelated to the patients.

## Results

### Study cohort

Four hundred and forty-nine adults undergoing elective colorectal surgery at the Department of Surgery, Umeå University Hospital, from March 2013 to April 2017 were eligible for inclusion. Fifteen patients were excluded; four because of postoperative confusion, five because they were intubated and treated in a respirator after surgery, and six because the NRS had not been recorded on any of 4 days. Seven patients, four colonic resections, and three anterior resections were converted from minimally invasive surgery to open surgery, and they all received an epidural catheter immediately after surgery for postoperative analgesia. Converted patients were regarded as open surgery in the analysis since they were given the same pre-medication, and all of them received an epidural catheter immediately after surgery for postoperative analgesia.

The analysis was based on the 434 included patients. The majority had undergone colorectal surgery due to cancer (90%). Baseline characteristics are presented in Table 1.

**Table 1:** Baseline characteristics

Age, mean (SD), years	69.5 ± 11.9
BMI, mean (SD), kg/m^2^	26.0 ± 4.6
Males, no. (%)	216 (49.8)
Females, no. (%)	218 (50.2)
ASA class 1, no. (%)	43 (10)
ASA class 2, no. (%)	242 (56)
ASA class 3–4, no. (%)	149 (34)
Diabetes mellitus, no. (%)	66 (15)
Non-smoker, no. (%)	403 (93)
Current smoker, no. (%)	12 (2.8)
Stopped smoking because of surgery, no. (%)	9 (2.0)
Colorectal cancer, no. (%)	390 (90)
Open colonic resection, no. (%)	169 (39)
Minimal colonic resection, no. (%)	77 (18)
Open anterior resection, no. (%)	69 (16)
Minimal anterior resection, no. (%)	26 (6)
Open abdominal perineal excision, no. (%)	72 (16)
Minimal abdominal perineal excision, no. (%)	21 (5)

*BMI* body mass index, *ASA* American Society of Anaesthesiologists, *SD* standard deviation

### Postoperative pain after colorectal surgery

**Fig. 1:**
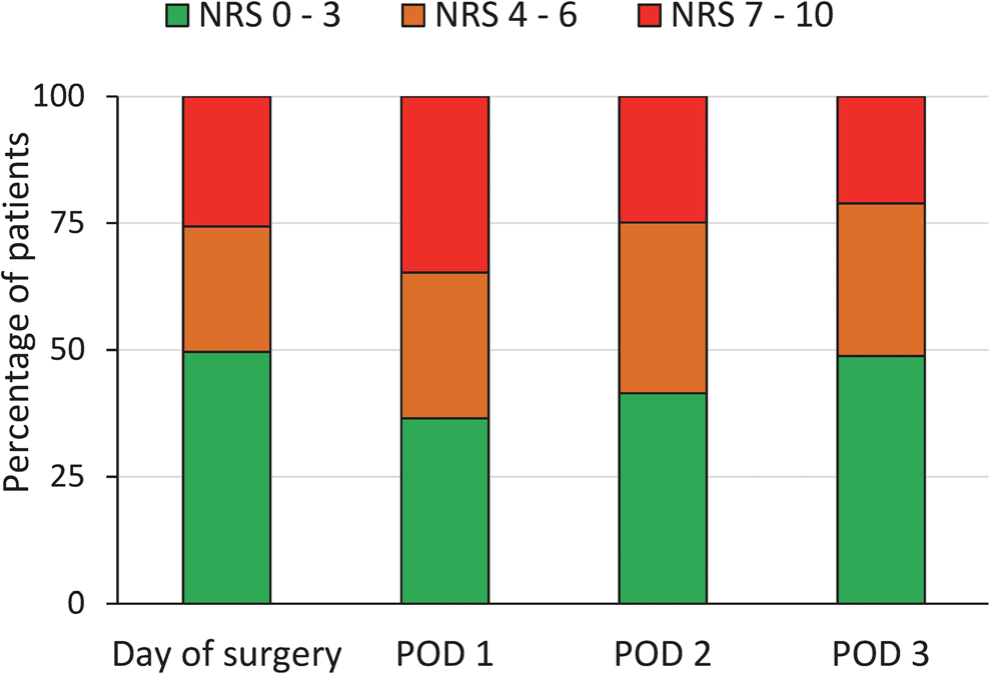
Distribution of pain score (NRS 0–10) on postoperative days 0–3. NRS, numeric rating scale; POD, postoperative day

**Fig. 2:**
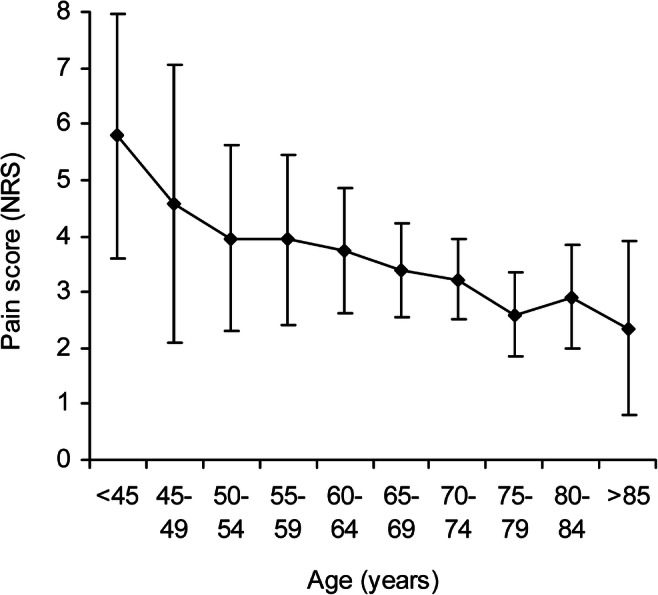
Age and pain score (NRS) on day of surgery, mean, and 95% confidence interval. NRS, numeric rating scale

Half the patients experienced moderate to severe pain (NRS > 4) on the day of surgery, followed by 64% on postoperative day 1, 59% on day 2, and 51% on day 3 (Fig.1). Patients younger than 45 years of age had more pain on the day of surgery compared with patients older than 75 with a mean NRS of 5.8 (95% CI 3.6 to 8.0) vs. 2.6 (95% CI 1.9–3.4) respectively, *P* = 0.01 (Fig. 2).

**Fig. 3:**
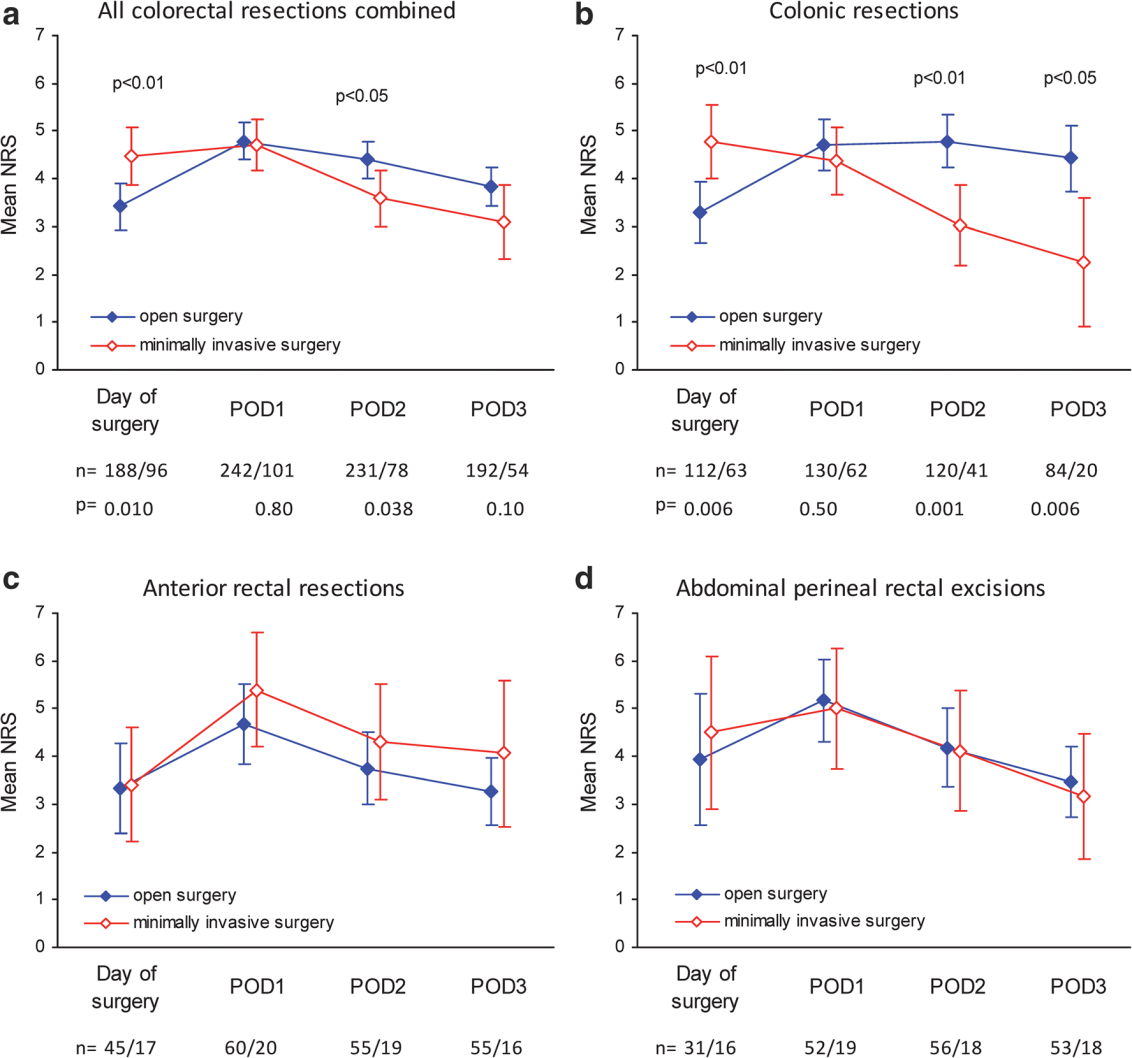
Mean and 95% confidence interval of pain score (NRS) during 4 postoperative days. **a** After all colorectal resections combined. **b** After colonic resections. **c** After abdominal rectal resections. **d** After abdominal perineal rectal excision. POD, postoperative day

Patients undergoing minimally invasive surgery vs. open surgery had more pain on the day of surgery and scored a mean of 4.5 (95% CI 3.9 to 5.1) vs. 3.4 (95% CI 2.9 to 3.9) respectively on the NRS, *P* < 0.001. On postoperative day 2, patients undergoing minimally invasive surgery had less pain compared with open surgery, 3.6 (95% CI 3.0 to 4.2) vs. 4.4 (95% CI 4.0 to 4.8) respectively, *P* = 0.038, while there was no difference in pain on postoperative days 1 and 3 (Fig. 3a). After minimal colonic resection vs. open surgery, patients had more pain expressed on the NRS on the day of surgery of 4.8 (95% CI 4.0 to 5.6) vs. 3.3 (95% CI 2.9 to 3.9) respectively (*P* = 0.006), while they had less pain on postoperative day 2 of 3.0 (95% CI 2.2 to 3.9) vs. 4.8 (95% CI 4.2 to 5.4) respectively (*P* = 0.001) and postoperative day 3 of 2.3 (95% CI 0.9 to 3.6) vs. 4.4 (95% CI 3.8 to 5.1) respectively (*P* = 0.006) (Fig. 3b). There was no significant difference in pain after minimally invasively vs. open anterior rectal resection and abdominal perineal rectal excision (Fig. 3c, d).

**Fig. 4:**
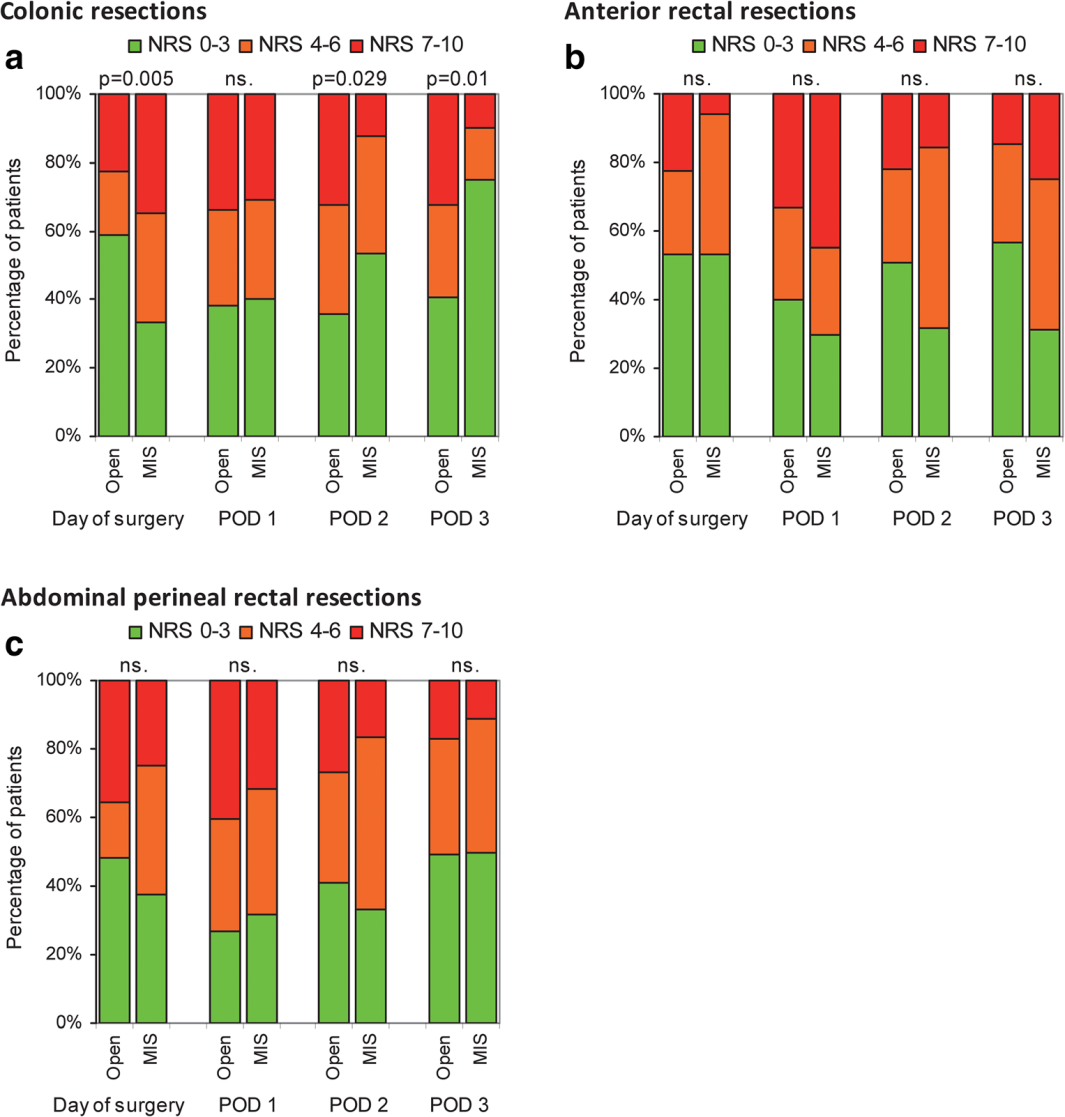
**a** Colonic resections. Categorical differences in pain between open and minimally invasive surgery. MIS, minimally invasive surgery; POD, postoperative day. **b** Anterior rectal resections. Categorical differences in pain between open and minimally invasive surgery. MIS, minimally invasive surgery; POD, postoperative day. **c** Abdominal perineal rectal excisions. Categorical differences in pain between open and minimally invasive surgery. MIS, minimally invasive surgery; POD, postoperative day

The interindividual pain response to surgery was large. Postoperative pain ranged from 0 to 10 after both minimally invasive and open colon and rectal surgery, despite adherence to the same pain management protocol (Fig. 4a–c).

### Postoperative course

A complication of any kind, including surgical, infectious, respiratory, and heart complications, was recorded in 239 patients (55%) within 30 days after surgery. A severe complication defined as Clavien-Dindo 3b or more occurred in 44 patients (10%) and 20 patients (4.6%) had an anastomotic leakage. The mean and standard deviation (SD) length of stay was 9 (9) days. On postoperative day 1, mean (standard deviation) high-sensitivity CRP was 73 (47) followed by 161(86) and 137 (79) on postoperative days 2 and 3.

### Risk factors for postoperative pain

#### Univariable analysis

In unadjusted analysis, age, diabetes mellitus, undergoing surgery for cancer, minimal vs. open surgery, having any complication, and CRP were significantly related to the NRS on any postoperative day (Table 2). There was no association between postoperative pain and gender, BMI, smoking, ASA class, preoperative chemo- or radiotherapy, or length of hospital stay.

**Table 2:** Unadjusted linear regression analysis on factors for maximum pain (NRS) on postoperative days 0–3

	Day of surgery		POD 1		POD 2		POD 3	
	Coeff (95% CI)	*p* value	Coeff (95% CI)	*P* value	Coeff (95% CI)	*P* value	Coeff (95% CI)	*P* value
Female vs. male	− 0.2 (− 1.0 to 0.6)	0.582	− 0.6 (− 1.2 to 0.04)	0.067	0.1(− 0.5 to 0.8)	0.713	0.4 (− 0.4 to 1.1)	0.319
Age (10 years)	− 0.7 (− 1.0 to − 0.3)	< 0.001	− 0.6 (− 0.9 to − 0.3)	< 0.001	− 0.4 (− 0.7 to − 0.1)	0.013	− 0.2 (− 0.5 to 0.1)	0.285
BMI (1 unit)	0.0 (− 0.1 to 0.1)	0.557	0.0 (− 0.1 to 0.1)	0.623	0.0 (− 0.04 to 0.1)	0.372	0.0 (− 0.1 to 0.1)	0.548
Current smoker*	− 0.4 (− 3.3 to 2.6)	0.810	0.4 (− 1.9 to 2.7)	0.732	0.6 (− 1.3 to 2.5)	0.513	1.9 (− 0.5 to 4.2)	0.123
ASA class 2 vs. 1	0.2 (−1.1 to 1.4)	0.800	1.0 (− 0.4 to 1.7)	0.217	0.5 (− 0.7 to 1.6)	0.421	− 0.1 (− 1.4 to 1.3)	0.930
ASA class 3–4 vs. 1	− 0.4 (− 1.7 to 1.0)	0.584	0.4 (− 0.7 to 1.5)	0.440	0.8 (− 0.4 to 2.0)	0.167	0.8 (− 0.7 to 2.2)	0.290
Diabetes mellitus*	− 1.5 (− 2.7 to − 0.4)	0.009	− 0.4 (− 1.3 to 0.6)	0.456	− 0.1 (− 1.0 to 0.8)	0.779	0.2 (− 0.8 to 1.2)	0.716
Cancer*	− 0.7 (− 1.9 to 0.5)	0.261	− 0.7 (− 1.7 to 0.3)	0.191	− 0.2 (− 1.3 to 0.9)	0.723	− 1.2 (− 2.5 to − 0.01)	0.048
Chemotherapy*	− 1.2 (− 2.8 to 0.5)	0.174	0.3 (− 1.0 to 1.6)	0.649	− 0.3 (− 1.6 to 0.9)	0.585	0.1 (− 1.1 to 1.3)	0.864
Radiotherapy*	0.2 (− 0.7 to 1.1)	0.680	0.4 (− 0.3 to 1.1)	0.257	0.0 (− 0.7 to 0.7)	0.957	− 0.3 (− 0.1 to 0.4)	0.409
Length of stay (> 10 days)	− 0.6 (− 1.4 to 0.3)	0.200	0.3 (− 0.4 to 1.0)	0.420	0.5 (− 0.2 to 1.2)	0.140	0.8 (− 0.0 to 1.5)	0.054
Clavien-Dindo > 3b*	0.1 (− 1.3 to 1.4)	0.917	− 0.1 (− 1.2 to 1.0)	0.828	− 0.4 (− 1.6 to 0.7)	0.448	− 1.4 (− 2.6 to − 0.3)	0.017
Anastomotic leakage*	− 1.2 (− 3.1 to 0.8)	0.234	− 0.4 (− 2.0 to 1.3)	0.668	0.1 (− 1.4 to 1.6)	0.935	0.5 (− 1.5 to 2.4)	0.646
Any complication	0.3 (− 0.5 to 1.0)	0.525	0.5 (− 0.2 to 1.1)	0.154	1.1 (0.4 to 1.8)	0.001	0.6 (− 0.2 to 1.3)	0.124
CRP (10 units)			− 0.15 (− 0.26 to − 0.04)	0.007	0.00 (− 0.01 to 0.01)	0.875	0.07 (0.02 to 0.12)	0.008
Minimal colonic vs. open colonic	1.5 (0.4 to 2.5)	0.005	− 0.3 (− 1.2 to 0.6)	0.502	− 1.8 (− 2.8 to − 0.7)	< 0.001	− 2.2 (− 3.6 to −0.8)	0.003
Minimal AR vs. open AR	0.1 (− 1.8 to 1.9)	0.933	0.7 (− 0.8 to 2.3)	0.347	0.6 (− 1.0 to 2.1)	0.464	0.8 (− 0.8 to 2.4)	0.334
Minimal APE vs. open APE	0.6 (− 1.4 to 2.6)	0.577	− 0.2 (− 1.8 to 1.4)	0.831	− 0.1 (− 1.6 to 1.5)	0.932	− 0.3 (− 1.9 to 1.2)	0.698

*Coefficient denotes the difference between the NRS and the referent. Yes vs. no

*Coeff* coefficient, *CI* confidence interval, *POD* postoperative day, *AR* anterior resection, *APE* abdominal perineal excision

#### Multivariable analysis

Adjusted linear regression analysis revealed that age, diabetes mellitus, any complication, and open vs. minimally invasive surgery were independent factors associated with a higher or lower NRS (Table 3). Pain was reduced with increasing age on postoperative days 0–2, while the NRS was reduced by 0.7 units per 10 years on the day of surgery (95% CI 0.5 to 0.9, *P* < 0.001) (Table 3, Fig. 2), thereby indicating that young subjects suffered from more pain than older subjects. On the other hand, patients with diabetes mellitus reported less pain by a mean of − 1.3 NRS (95% CI − 2.4 to − 0.2, *P* = 0.025) on the day of surgery. Having any complication after surgery was independently related to more pain on postoperative day 2 by 1.1 NRS (95% CI 0.2 to 2.0, *P* = 0.02). High CRP levels on postoperative day 1 were related to less pain on that day by 0.15 NRS/10 units of CRP (95% CI − 0.26 to − 0.04, *P* = 0.008), while high CRP on postoperative day 3 was related to more pain by 0.07/10 units of CRP (95% CI 0.01 to 0.12, *P* = 0.038).

**Table 3:** Adjusted linear regression analysis on factors for maximum pain (NRS) on postoperative days 0–3

	Day of surgery		POD 1		POD 2		POD 3	
	Coeff (95% CI)	*P* value	Coeff (95% CI)	*P* value	Coeff (95% CI)	*P* value	Coeff (95% CI)	*P* value
Female vs. male	− 0.3 (− 1.1 to 0.4)	0.380	− 1.0 (− 2.1 to − 0.0)	0.049	0.1 (− 0.8 to 1.1)	0.809	0.6 (− 0.3 to 1.4)	0.177
Age (per 10 years)	− 0.7 (− 1.0 to − 0.3)	< 0.001	− 0.8 (− 1.2 to − 0.3)	< 0.001	− 0.2 (− 0.6 to 0.2)	0.320	− 0.2 (− 0.5 to 0.2)	0.410
Diabetes mellitus*	− 1.3 (− 2.4 to − 0.2)	0.025	− 0.2 (− 1.7 to 1.3)	0.832	− 0.7 (− 1.9 to 0.5)	0.222	− 0.3 (− 1.4 to 0.8)	0.587
Cancer*	− 0.0 (− 1.2 to 1.2)	0.957	1.2 (− 0.4 to 2.7)	0.133	0.0 (− 1.5 to 1.5)	0.963	− 0.5 (− 2.1 to 1.2)	0.579
Any complication*	0.4 (− 0.3 to 1.2)	0.273	− 0.1 (− 1.2 to 1.0)	0.820	1.1 (0.2 to 2.0)	0.020	0.8 (− 0.1 to 1.6)	0.081
CRP (per 10 units)			− 0.15 (− 0.26 to − 0.04)	0.008	− 0.02 (− 0.07 to 0.04)	0.518	0.07 (0.01 to 0.12)	0.014
Minimal colonic vs. open colonic	1.6 (0.6 to 2.6)	0.002	− 0.6 (− 2.0 to 0.9)	0.450	− 2.3 (− 3.9 to − 0.7)	0.004	− 1.7 (− 3.3 to − 0.1)	0.038
Minimal AR vs. open AR	0.2 (− 1.6 to 1.9)	0.858	2.3 (− 0.4 to 5.0)	0.092	1.0 (− 1.9 to 4.0)	0.480	1.0 (− 0.8 to 2.7)	0.280
Minimal APE vs. open APE	0.6 (− 1.3 to 2.6)	0.537	− 0.6 (− 3.2 to 2.0)	0.638	− 1.2 (− 3.7 to 1.3)	0.346	− 0.0 (− 1.8 to 1.7)	0.978

*Coefficient denotes the difference between the NRS and the referent. Yes vs. no. *Coeff* coefficient, *CI* confidence interval, *POD* postoperative day, *AR* anterior resection, *APE* abdominal perineal excision

Patients undergoing minimally invasive colonic surgery had more pain than patients undergoing open surgery on the day of surgery by 1.6 NRS (95% CI 0.6 to 2.6, *P* = 0.002), while those undergoing open surgery had more pain on postoperative day 2 by 1.5 NRS (95% CI 0.4 to 2.5, *P* = 0.006) and day 3 by 1.9 NRS (95% CI 0.4 to 3.3, *P* = 0.011) (Table 3). There was no significant difference in postoperative pain on any day between open anterior resection vs. minimally invasive surgery, or between open abdominal perineal excision and minimally invasive surgery (Table 3).

### Sensitivity analysis

Seven patients were converted from minimally invasive surgery to open surgery and are regarded as open surgery in the above analysis. The results did not change when these seven converted patients were excluded.

## Discussion

This cohort study shows that more than half the patients experienced moderate to severe pain on each postoperative day after elective colorectal surgery, despite adhering to an ERAS perioperative program including long- and short-acting opioids on demand. Younger patients and patients with any complication experienced more pain after surgery, while patients with diabetes mellitus experienced less pain. The results also show that patients undergoing minimally invasive surgery reported a high degree of postoperative pain, comparable with open surgery. Another finding was a large interindividual variability in pain intensity after each surgical modality, despite the same analgesic regimen. This study highlights the need for more effective pain management protocols for individuals undergoing colorectal surgery, including both open and minimally invasive procedures, particularly in young individuals.

Minimally invasive surgery has been reported to both reduce postoperative pain and morbidity and shorten the length of hospital stay [22]. In the present study, patients undergoing minimally invasive colonic surgery had more pain on the day of surgery and less on postoperative day 2 and 3 compared with patients undergoing open colonic surgery. Less pain after open surgery on the day of surgery is clearly due to epidural analgesia given for 1 day after open surgery, while more pain on postoperative days 2 and 3 is likely due to the removal of the epidural analgesia. As a result, patients undergoing minimally invasive colonic surgery are in need of more analgesia on the day of surgery, while patients undergoing open colonic surgery would probably benefit from epidural analgesia for 3 days instead of 1. Epidural analgesia for 3 days was given to patients undergoing open rectal surgery which could explain why there was no difference compared with minimally invasive rectal surgery. According to our results, there is a need for better pain control after colorectal surgery, except on postoperative days 2 and 3 after minimally invasive colonic surgery. Transversus abdominis plane blockade is another option to reduce postoperative pain after minimally invasive surgery and is recommended in recent ERAS guidelines [17, 23, 24].

Younger patients experienced more pain after surgery. The pain score was reduced by as much as 0.7 NRS units per 10 years on average on the day of surgery and remained significant on postoperative days 1 and 2, despite the fact that patients were given extra analgesia on demand. Similarly, Thige et al. found that younger age was related to more postoperative pain, on average by a half NRS unit per 10 years, when retrospectively analyzing postoperative pain after various operations during 24 h after surgery [12]. A recent meta-analysis by Lautenbacher et al. investigated pain perception and pain tolerance with age [25]. They showed that mental pain perception is not affected with age, but a loss of pain sensitivity occurs with an increase in pain thresholds in older adults. The renal clearance of opioids is also reduced with increasing age and may therefore contribute to less pain in older subjects [26]. The present results indicate that there is already a need for more analgesia in young subjects on the day of surgery.

Recent ERAS guidelines for postoperative analgesia after colorectal surgery state that “the key is to avoid opioids and apply multimodal analgesia in combination with epidural analgesia (in open surgery) when indicated” [17]. To the ERAS recommendation, we suggest adding that young patients need more analgesia and that analgesia should be individualized, since the amount of postoperative pain is difficult to predict.

Diabetes mellitus was independently associated with less postoperative pain, which is a new finding. Rajamäki et al. observed that diabetes mellitus was a risk factor for persistent pain after hip or knee replacement [27]. Several factors, including surgery on different organs, may account for the differing results. About 30% of diabetic patients develop neuropathic pain [28]. However, diabetic neuropathy is also related to reduced sensory input, which could explain why our patients with diabetes experienced less postoperative pain [29].

Female gender and a high ASA class have been suggested as causes of postoperative pain in general [8–12]. In this study, neither female gender nor ASA class was significantly related to pain. As a result, our findings do not support the hypothesis that gender and ASA class affect pain after abdominal surgery. As many as 55% of the present patients had some kind of complication, and having any complication was related to more pain on postoperative day 2. This supports the importance of reducing all complications, even mild, which is a goal of the ERAS perioperative program.

One strength of the present study is the analysis of a homogeneously treated cohort of patients undergoing colonic or rectal surgery during 4 days after surgery, with only a few drop-outs. The results are derived from a single center, which is a limitation. Another weakness of the study is the lack of data relating to personal pain thresholds, including depression and anxiety [8, 9].

In conclusion, the majority, and young patients in particular, experience moderate to severe pain after open and minimally invasive colorectal surgery, despite following the ERAS perioperative program for analgesia, including epidural analgesia after open surgery, and local anesthesia and opioids after minimally invasive surgery. There is a need for effective and individualized analgesia after colorectal surgery, since the individual pain response to surgery is difficult to predict.
